# Evaluation of shear bond strength of orthodontic ceramic and metal brackets with zirconia: effects of adhesive systems and storage conditions

**DOI:** 10.1007/s00784-026-06783-1

**Published:** 2026-03-18

**Authors:** Aya A. Salama, Karim A. Shehab, Alaa Mohamed Naguib

**Affiliations:** 1 Associate Professor of Prosthodontics, Faculty of Dentistry, October University for Modern Sciences and Arts, 6th October City, Egypt; 2 Lecturer of Orthodontics, Faculty of Dentistry, October University for Modern Sciences and Arts, 6th October City, Egypt; 3 Department of Preventive Dental Sciences, College of Dentistry, Gulf Medical University, Ajman, UAE; 4 Lecturer of Prosthodontics, Faculty of Dentistry, October University for Modern Sciences and Arts, 6th of October City, Egypt

**Keywords:** Shear bond strength, Metal bracket, Ceramic bracket, 5YTZP zirconia, HEMA, Storage

## Abstract

**Objective:**

The current study assessed the shear bond strength of two orthodontic bracket materials to zirconia using two bonding approaches and two storage settings.

**Methods:**

Two bonding strategies were selected to bond metal and ceramic brackets to zirconia samples. Before being tested for Shear Bond Strength [SBS], samples were either subjected to 5,000 thermocycles or kept in water for 24 h. Samples were divided into 8 Groups [*n* = 10] represented as **Group 1**: Ceramic Brackets + HEMA-Free Bonding System + Water Storage, **Group 2**: Ceramic Brackets + HEMA-Containing Bonding System + Water Storage, **Group 3**: Metal Brackets + HEMA-Free Bonding System + Water Storage, **Group 4**: Metal Brackets + HEMA-Containing Bonding System + Water Storage, **Group 5**: Ceramic Brackets + HEMA-Free Bonding System + Thermocycling, **Group 6**: Ceramic Brackets + HEMA-Containing Bonding System + Thermocycling, **Group 7**: Metal Brackets + HEMA-Free Bonding System + Thermocycling, **Group 8**: Metal Brackets + HEMA-Containing Bonding System + Thermocycling. Several independent groups were compared using the Kruskal-Wallis test, and two independent groups were assessed using the Mann-Whitney U test. A P-value < 0.05 was deemed significant.

**Results:**

Shear bond strength was significantly greater in ceramic than in metal [5.9 ± 2.6 vs. 4.9 ± 2.3, *p* = .026] and in 2-Hydroxyethyl methacrylate HEMA-containing systems compared to HEMA-free systems [6.1 ± 2.3 vs. 4.8 ± 2.4, *p* = .034]. Water storage settings also yielded higher bond strength than thermocycling conditions [6.9 ± 1.8 vs. 3.9 ± 2.2, *p* < .001].

**Conclusion:**

The findings indicate that shear bond strength was significantly influenced by bracket material and adhesive system to zirconia. Additionally, storage settings play a crucial part in shear bond strength.

**Clinical relevance:**

Developing effective bonding techniques for orthodontic brackets and zirconia ceramic materials remains a major clinical obstacle.

## Introduction

The increasing demand for dental aesthetics has resulted in an increase in the number of people seeking orthodontic treatment, including those who have previously had fixed prosthetic restorations [1, 2]. The bonding of orthodontic brackets to teeth is an important feature of fixed orthodontic appliances [3].

One clinical problem with adult orthodontic therapy is achieving the highest possible bonding force between orthodontic brackets and prosthetic surfaces, particularly zirconia [4]. The average forces needed for tooth movement have been estimated by researchers to be between 70 and 120 g. In order to promote clinical orthodontic movement, the claimed minimum bonding strength between orthodontic brackets and bonding substrate is 6–8 megapascals (MPa) [5]. Therefore, the strength of the bond at the bracket-adhesive-prosthesis must sustain orthodontic forces without breaking and this is challenging with zirconia ceramic material [6].

Zirconia is now widely used in dentistry for fixed prostheses [7]. Its superior mechanical strength distinguishes it from other common ceramic materials [8]. Furthermore, zirconia has good biocompatibility, visually appealing characteristics, and strong corrosion resistance [9]. By mixing zirconia [ZrO2] with yttria [Y2O3], yttrium-stabilized zirconia [YSZ] is produced; it exhibits improved molecular stability [10]. Yttria concentration in dental YSZ normally ranges between 3 and 6 mol% [11]. Although zirconia is difficult to acid-etch and silanize, which compromises the adhesion process. It is still difficult to establish a reliable bond between it and resin cement, despite its microstructural properties, chemical inertness, and biocompatibility [12, 13].

A generation of 5 mol% Yttria-stabilized Tetragonal Zirconia Polycrystal [5YTZP] zirconia, with a cubic phase of 10–50% yttria-stabilized zirconia, has been developed especially for aesthetic restorations. It offers a seamless translucency transition by eliminating the noticeable colour layer [14]. On the other hand, research is still being done to evaluate their effectiveness, especially in terms of bonding properties to orthodontic brackets [15, 16].

Orthodontic brackets come commonly in two types: metal and ceramic [17]. Because of their stark colour contrast with natural teeth, metal brackets which are usually composed of medical-grade stainless steel are frequently less preferred by many patients [18]. On the other hand, because of their cosmetic resemblance to tooth shades, monocrystalline or polycrystalline alumina ceramic brackets are recommended [19]. In contrast to conventional metal brackets, ceramic brackets have a weaker adhesion to enamel and ceramic surfaces, despite their esthetic appeal [20, 21]. Additionally, during debonding, ceramic brackets can result in irreparable tooth injury and are prone to fracture [22].

The bonding strength between ceramic and metal brackets and zirconia is mostly determined by the bonding agent selection, particularly 2-Hydroxyethyl methacrylate [HEMA] including both HEMA-containing and HEMA-free formulations [23]. In recognition of its hydrophilic properties, which improve wetting and penetration into the substrate, 2-hydroxyethyl methacrylate (HEMA) is a commonly used monomer in dental adhesives [24]. Bonding compounds containing HEMA may improve adhesion in the context of metal and ceramic brackets by improving penetration into the ceramic matrix and interacting chemically with the metal surface [25]. But over time, HEMA might also result in more water absorption, which could weaken bonds by causing hydrolytic breakdown. When moisture is present, this issue is very problematic as it may shorten the bond’s lifespan, potentially leading to clinical failure [26].

The effectiveness of adhesion of metal and ceramic brackets to ceramic surfaces.

is greatly impacted by moisture and aging [27]. Resin cements may hydrolyze when exposed to moisture, gradually weakening the bond [28]. Thermocycling causes expansion and contraction that could further jeopardize the bond’s stability by simulating the thermal changes found in the oral environment [29]. To ensure long-lasting orthodontic effectiveness and optimize adhesive techniques, it is imperative to comprehend these aspects.

Improving the bonding ability between zirconia and orthodontic brackets could be very beneficial to patients in order to accomplish the long-term orthodontic objectives [30]. Only a small number of previous studies have looked at how different bonding agents can affect the longevity between zirconia and different bracket materials [31].

With regard to their use on metal or ceramic brackets and zirconia, this research attempted to assess the bonding efficacy of two distinct bonding agents under two storage settings either 24 h distilled water or 5,000 thermal cycles. The following were the null hypotheses: [1] there is no significant difference in the shear bond strength of ceramic brackets compared to metal brackets when bonded to zirconia samples [2]. There is no significant difference in the shear bonding strength between HEMA-free bonding systems and HEMA-containing bonding systems when zirconia is bonded to orthodontic brackets [3]. The shear bond strength of orthodontic brackets bonded to zirconia that are kept in distilled water for 24 h does not differ significantly from that of brackets that are subjected to thermocycling for 5,000 cycles.

## Materials and methods

Ethical approval was obtained from the institution ethical committee with Reference no. 1945. The sample size was determined using a prior study by Hu et al. 2024 [30]. With 80% power and at the 5% significant level, and with a 1.1 effect size, a total of eight samples were determined to be the smallest sample size. To account for roughly 25% dropouts, this was changed to *n* = 10, making 80 samples total the final sample size. G*Power calculated the sample size (version 3.1.9.2; Germany).

### Sample Preparation

An Isomet 4000 linear precision saw (Buehler Ltd., Lake Bluff, IL) was used to precision Sect. 80 flat surface rectangular sampless made of zirconia (Ceramill Zolid fx; Amann Girrbach, Austria) that were 10 mm by 6 mm by 2 mm for the investigation. Based on the type of orthodontic brackets bonded to zirconia, the bonding system used, and the storage environment, Eight experimental groups (*n* = 10) were randomly assigned to these samples:

- **Group 1**: Ceramic Brackets + HEMA-Free Bonding System + Distilled Water Storage (24 h) [C-HF-W]
- **Group 2**: Ceramic Brackets + HEMA-Containing Bonding System + Distilled Water Storage [24 h] [C-HC-W]
- **Group 3:** Metal Brackets + HEMA-Free Bonding System + Distilled Water Storage (24 h) [M-HF-W]s
- **Group 4:** Metal Brackets + HEMA-Containing Bonding System + Distilled Water Storage (24 h) [M-HC-W]
- **Group 5:** Ceramic Brackets + HEMA-Free Bonding System + Thermocycling [C-HF-T]
- **Group 6:** Ceramic Brackets + HEMA-Containing Bonding System + Thermocycling [C-HC-T]
- **Group 7:** Metal Brackets + HEMA-Free Bonding System + Thermocycling [M-HF-T]
- **Group 8: **Metal Brackets + HEMA-Containing Bonding System + Thermocycling [M-HC-T].

As endorsed by the manufacturer, zirconia samples were sintered. On a rotating polishing machine (Jean Wirtz, Charlottenstr. 73, Düsseldorf, Germany); silicon carbide (SiC) abrasive sheets with a fine silicon carbide 600 grit (Struers, Copenhagen, Denmark) were applied under copious water by a single trained operator for 1 min. The rotational speed of the polishing machine was set at approximately 150 RPM to polish the sintered zirconia rectangular samples [32]. After being cleaned in an ultrasonic bath and allowed to dry, the samples were sandblasted for 60 s with 125 μm aluminum oxide particles at a one centimeter distance, 45 degrees angle and 2.5 bar of pressure. After that, all samples were submitted to gentle air drying and ultrasonic cleaning in distilled water for five minutes.

## Brackets bonding approach

Transbond XT Orthodontic Adhesive Primer, a HEMA-free bonding method, was applied to half of the samples intended for the metal and ceramic brackets (3 M Unitek, United States). On the zirconia, a single drop of primer was applied. Light curing is done (1400 mW/cm2, D-Light Pro, GC Dental, and Japan) for 20 s after the primer has been gently blown dry for 15 s to create a thin, even coat. The other half was treated with HEMA-containing bonding system Single Universal Bond (3 M ESPE, United States). Apply the adhesive to the bracket base and zirconia surface. Lightly cure the bond (1400 mW/cm2, D-Light Pro, GC Dental and, Japan) for 20 s after gently blow-drying it for 15 s to create a thin, even layer.

The ceramic samples were bonded to the metal brackets (Ormco Corp., Glendora, California, USA) and ceramic brackets (Ormco Corp., Glendora, California, USA). An upper incisor brackets were bonded using the light-cure adhesive composite Transbond XT (3 M Unitek, USA). The resin adhesive was placed over the zirconia disk on the bracket base. Extra material was then forced out by hand. A periodontal probe was used to remove any excess. A (1400 mW/cm2, D-Light Pro, GC Dental, Tokyo, Japan) was used for 20 s to polymerise the adhesive resin cement. Table 1 provides information about the chemical composition of the materials used in this study.

**Table 1:** The chemical composition of the materials used

Material	Chemical Composition
Transbond XT Orthodontic Adhesive Primer [3 M Unitek, United States]	Triphenylantimony, Bis-GMA and TEGDMA, CQ, and DMAEMA
Single Bond Universal [3 M ESPE, United States]	HEMA,10-MDP, polyalkenoic acid copolymer, dimethacrylate resins, filler, ethanol, water, initiators, and silane
Transbond XT adhesive paste resin cement [3 M Unitek]	Bis-GMA, Bis-EMA quartz, DMAEMA, silicon dioxide, and canforquinone
Ceramill Zolid fx, sintering temperature in Ceramill Therm at 1450 °C [Amann Girrbach, Koblach, Austria/ 1904000]	Zirconium oxide, Yttrium oxide 8.5–9.5%., Hafnium oxide HfO2 < 5%, Silicon oxide, Aluminum oxide Al2O2, other oxides < 1%

HEMA, 2-hydroxyethyl methacrylate; TEGDMA, triethyleneglycol dimethacrylate; Bis-GMA, bisphenol A-diglycidyl methacrylate; Bis-EMA, bisphenol A ethoxylated dimethacrylate; 10-MDP, 10- methacryloyloxydecyl dihydrogen phosphate; CQ, camphorquinone; DMAEMA, dimethylaminoethyl methacrylate

## Shear bond strength test

The samples were divided established on the storage method into two distinct groups. Each subgroup’s brackets were examined after half were stored in water at 37 °C for 24 h, and the remaining brackets were thermocyclically cycled 5,000 times between 5 °C and 55 °C in a thermocycling device (Thermocycler THE-1100; SD-mechatronik). This division allowed for a relative analysis of the effects of storage settings on the shear bond strength of the various bonding systems and different orthodontic brackets materials.

**Fig. 1:**
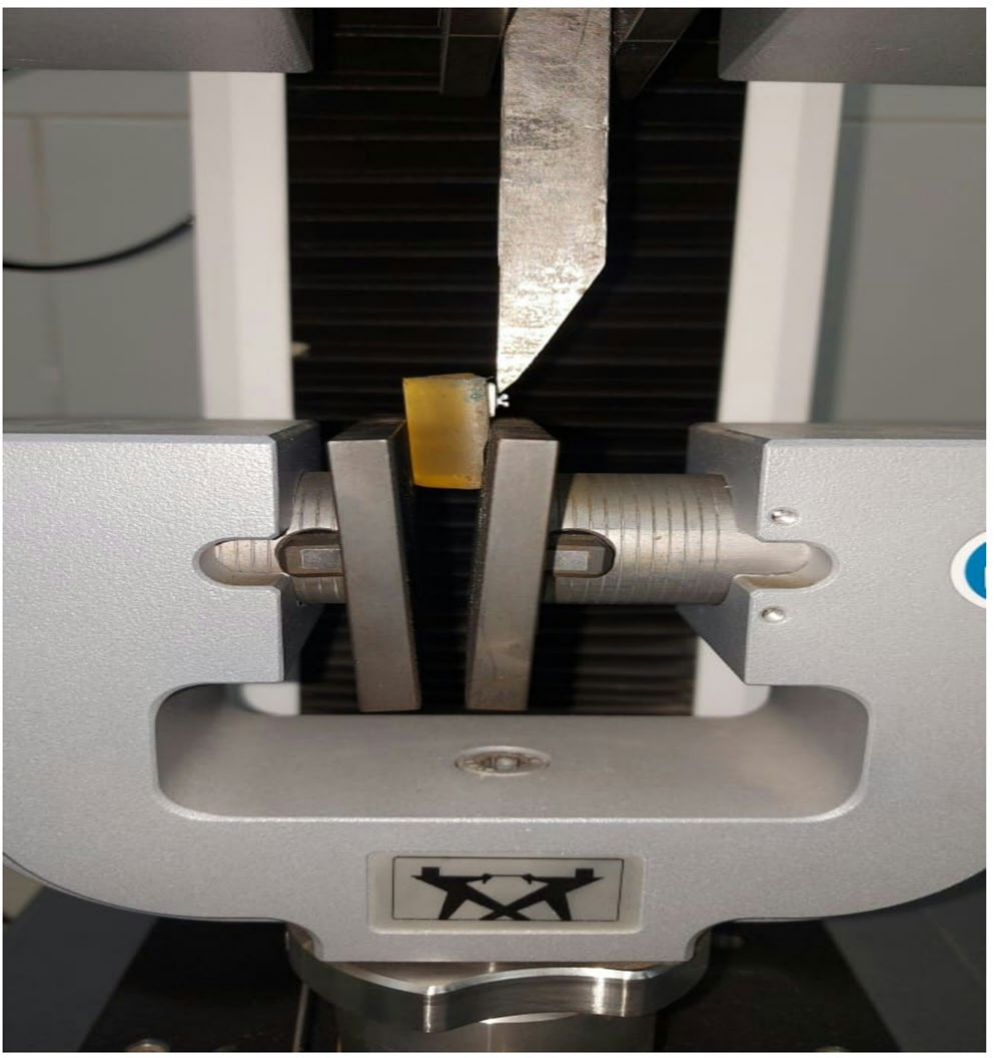
Shear bond strength test showing the loading blade positioned tangentially to the bracket base using Universal Testing Machine

A universal testing machine (Model 3345, Instron Industrial Products) with a knife-edge loading device applying a shear force at a cross-head speed of 1 mm/min applying a shear force parallel to the bonding interface was utilized to perform the SBS test, following a modified shear bond strength test design previously used in orthodontic studies [33, 34]. The triangular-section blade applied the load directly at the bracket base-adhesive interface to produce a shear force parallel to the bonding surface. This methodology allows for the isolation of shear stress but differs from the testing configuration described in the DIN 13990:2017 standard. According to the DIN standard, the load is typically applied via a metal blade in a manner that may introduce a tensile load component on the bracket base-adhesive interface, influencing the fracture mechanics differently than a pure shear setup [35, 36]. The SBS formulation was P [MPa] megapascals = F [N] Newtons / S, which is the bracket’s base surface area (10 mm^2^) **(**Fig. 1**)**.

### Statistical analysis

The datasets were analyzed using version 26 of the Statistical Package of Social Sciences (SPSS). To ascertain whether the data was normal, the Kolmogorov-Smirnov single-sample test was utilized. Numerical variables were displayed using the mean and standard deviation. Whereas the Mann-Whitney U test was used to compare two independent groups, the Kruskal-Wallis test was used to compare more than two independent groups. The Benferroni adjustment was formerly used to correct the p-value in a post hoc test. It was deemed significant if (*P*≤ .05).

## Results

**Fig. 2:**
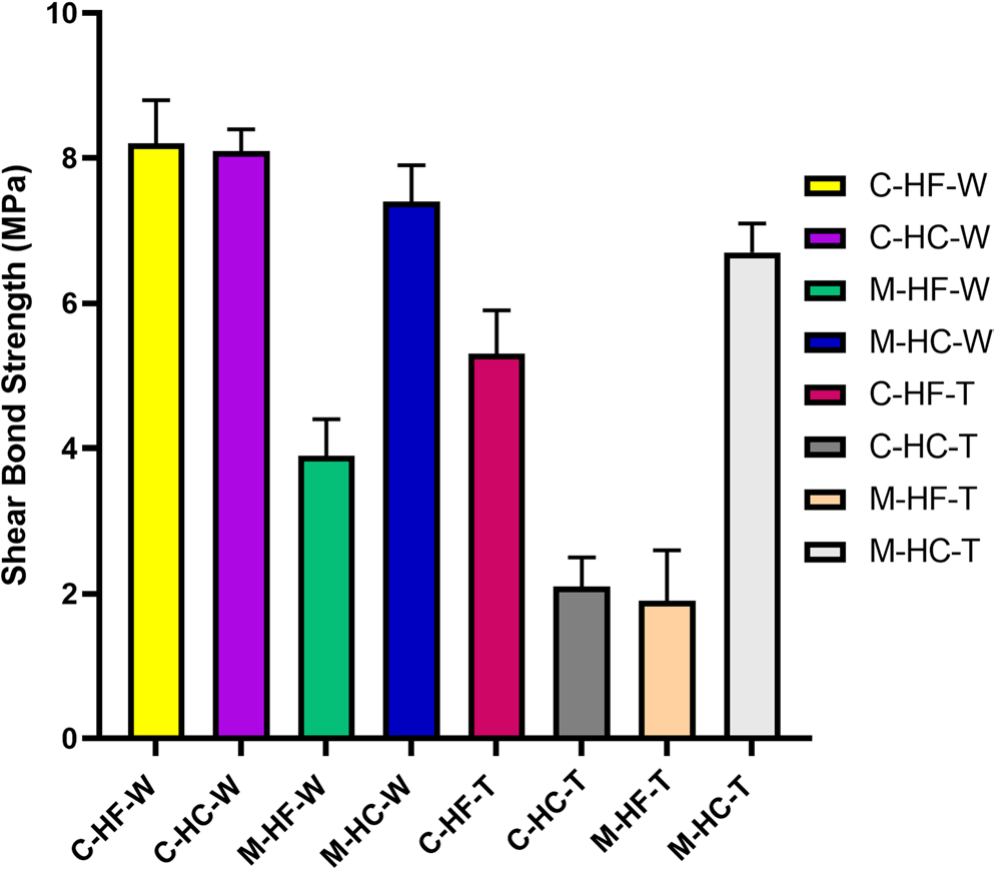
Comparison of shear bond strength [MPa] between groups

Table 2 summarizes the mean shear bond strength (SBS) values and standard deviations for each group. The differences in SBS (MPa) among the groups were statistically significant, with an overall p-value of < 0.001. Pairwise comparisons indicated that C-HF-W had a significantly higher shear bond strength than M-HF-W, C-HF-T, C-HC-T, and M-HF-T (adjusted p-values: 0.001, 0.033, < 0.001, < 0.001). However, no significant differences were found among M-HC-W, C-HC-W, M-HC-T, and C-HF-W. Additionally, C-HC-W showed greater shear bond strength compared to M-HF-W, C-HF-T, C-HC-T, and M-HF-T (adjusted p-values: 0.001, 0.037, < 0.001, < 0.001), with no significant differences among M-HC-T, M-HC-W, and C-HC-W. M-HC-W had higher shear bond strength than C-HC-T and M-HF-T (adjusted p-value < 0.001), but no significant difference was noted between M-HC-T, C-HF-T, and M-HC-W. No significant differences were observed among C-HC-T, M-HF-T, M-HC-T, and C-HF-T. C-HC-T showed lower shear bond strength than M-HC-T (adjusted p-value = 0.018), and M-HF-T had significantly lower shear bond strength compared to M-HC-T **(**Fig. 2**)** (adjusted p-value = 0.009).

**Table 2:** Comparison of shear bond strength [MPa] between groups

Groups	C-HF-W	C-HC-W	M-HF-W	M-HC-W	C-HF-T	C-HC-T	M-HF-T	M-HC-T	*p*-value
Shear Bond Strength [MPa]	8.2±.6a	8.1±.3a	3.9±.5b	7.4±.5a	5.3±.6b	2.1±.4bc	1.9±.7bc	6.7±.4a	**< 0.001**

Different small letters in the column indicate significant difference for shear bond strength (*p* < .001)

There was a statistically significantly higher shear bond strength (MPa) in ceramic than in metal (5.9 ± 2.6 versus 4.9 ± 2.3, p-value=0.026). There was a statistically significantly higher average shear bond strength (MPa) in HEMA-containing than in HEMA-free bonding system (6.1 ± 2.3 versus 4.8 ± 2.4, p-value=0.034). There was a statistically significantly higher shear bond strength (MPa) in water than in Thermocycling storage condition (6.9 ± 1.8 versus 3.9 ± 2.2, p-value < 0.001) **(**Table 3**)**.

**Table 3:** Comparison of shear bond strength [MPa] between metal versus ceramic brackets

Groups	Shear Bond Strength [MPa]	*p*-value
Metal [*n* = 40]	4.9 ± 2.3a	0.026
Ceramic [*n* = 40]	5.9 ± 2.6b	
HEMA-containing [*n* = 40]	6.1 ± 2.4a	0.034
HEMA free [*n* = 40]	4.8 ± 2.3b	
Water storage [*n* = 40]	6.9 ± 1.8a	< 0.001
Thermocycling[*n* = 40]	3.9 ± 2.2b	

Different small letters in the column (within each condition: types of brackets, HEMA free or not, and Thermocycling or not) indicate significant difference for shear bond strength (*p* < .001)

## Discussion

This research study aimed to compare the shear bond strength of two bonding systems using ceramic and metal brackets on zirconia after either water storage or thermocycling. Significant variations in shear bond strength [SBS] between groups were discovered, leading to the rejection of the null hypothesis, as there was significant difference in shear bond strength. The bonding strength of orthodontic brackets ought to range between 6 and 8 MPa for the best orthodontic performance [5].

All metal bracket groups, except for the M-HF-W and M-HF-T groups, obtained long-term shear bond strengths that were clinically acceptable. Following a 24-hour water storage period, the M-HF-W group—which served as a control for bonding brackets to zirconia—exhibited the lowest SBS. This indicates that bonding metal brackets primarily depends on the presence of the HEMA component; without it, bonding to zirconia was compromised and fell below clinical acceptance [23]. Additionally, upon thermocycling, the SBS OF M-HF-W considerably dropped, most likely as a result of mismatched coefficients of expansion and shrinkage, failing to achieve the necessary clinical shear bond strength.

As 10-MDP uses phosphate groups to promote chemical bonding with zirconia, the C-HC-W group, which used a universal HEMA-containing adhesive, demonstrated better bonding performance [37, 38]. In contrast, HEMA-free bonding agents utilize alternative monomers that provide increased hydrophobicity and reduced water sorption, which can enhance long-term bond stability to both metal and ceramic substrates [24, 25]. These HEMA-free formulations often demonstrate superior shear bonding strength and resistance to hydrolysis, particularly with 5YTZP zirconia ceramics, where the bonding interface must withstand significant occlusal forces. The absence of HEMA reduces the likelihood of bond degradation in moisture, thereby enhancing the longevity and reliability of restorations [30]. This explains why the C-HC-T group exhibited lower SBS compared to the C-HF-T group, which demonstrated durable bonding ability even after thermocycling.

SBS was significantly impacted by storage conditions. According to a study, adhesives containing HEMA continuously absorb water, which weakens the binding during polymerization [26]. After 5,000 cycles of thermocycling, the C-HC-T group showed a notable decrease in SBS, primarily as a result of water absorption. The adhesive may hydrolyze as a result, weakening the bonds. In contrast, the M-HC-T group’s SBS remained constant, indicating that it is resistant to water-induced breakdown, something that was not seen in previous research [39]. This suggests that the bonding system’s formulation and the metal brackets’ material properties both affect how resistant they are to age and moisture.

The M-HC-T group only slightly decreased SBS after thermocycling, compared to most other groups. A possible explanation is that elevated temperatures during resin settling might have facilitated radical mobility and polymerization, thereby improving shear bond stability; however, this remains a hypothesis requiring further confirmation but it matched the results of previous research [39]. The null hypothesis that storage conditions have no effect on hear bond strength is partially rejected by this discrepancy. Compared to samples that underwent thermocycling, samples that were merely in water for 24 h showed a notably higher bonding strength. This highlights how important it is to take environmental factors like moisture and temperature changes into account when assessing the long-term effectiveness of orthodontic bonding.

With the exception of M-HF-W, the SBS of ceramic and metal brackets was comparable following a 24-hour water storage period. However, there was a statistically significantly higher total shear bond strength (MPa) in ceramic than in metal brackets (p-value = 0.026).The null hypothesis that the shear bond strength of metal and ceramic brackets on zirconia would be equal was rejected by the ceramic brackets’ noticeably higher SBS after thermocycling. The C-HF-T group produced SBS values below the 6–8 MPa threshold widely cited for clinical acceptability in orthodontic bonding. This should be explicitly acknowledged, as it limits the clinical applicability of that bonding combination despite being comparatively higher than other thermocycled groups.

One possible explanation for this discrepancy lies in the inherent properties of the materials involved. Metal brackets typically have a smoother surface compared to ceramic brackets, which can impact the mechanical interlocking with the adhesive. The rougher surface of ceramic brackets may provide a greater surface area for the adhesive to bond, enhancing the mechanical retention and overall bond strength.

Additionally, the presence of the HEMA component in some bonding agents can affect the bond strength differently depending on the substrate. While HEMA-containing adhesives may promote strong bonding with ceramic materials due to chemical interactions, their performance with metal may be compromised if the light cannot penetrate adequately to cure the adhesive uniformly.

Moreover, the thermal expansion coefficients of the materials can create stresses at the bonding interface during temperature fluctuations, particularly during the bonding process and subsequent thermocycling. Metal brackets, which have different thermal properties compared to zirconia, may experience greater stress at the bond interface, leading to a reduction in SBS over time.

The findings suggest that the choice of adhesive and the properties of the bonding materials play a crucial role in achieving optimal bond strength. It is imperative to recognize that while metal brackets offer certain advantages, such as durability and ease of handling, their performance in bonding to zirconia may not be as reliable as that of ceramic brackets due to the factors mentioned above. This observation underscores the need for further investigation into adhesive systems specifically designed for bonding metal to zirconia, as well as the importance of considering the physical and chemical interactions at play in these bonding scenarios. The superior bond strength of ceramics may be attributed to the higher surface energy and micromechanical interlocking produced by surface treatment, which enhances resin penetration and silane coupling efficiency. This observation aligns with the surface chemistry mechanisms reported in recent zirconia bonding investigations [15, 30].

The importance of material selection in orthodontic applications is highlighted by the possibility that ceramic brackets may be affected by repeated heat stress and aging compared to their metal counterparts, yet they provide better aesthetic appeal and higher shear bond strength to zirconia [22]. Clinically, these findings suggest that optimizing surface treatment and bonding agent selection for zirconia restorations can minimize bracket failure during orthodontic therapy, improving treatment efficiency and reducing chairside time.

Regarding limitations, a larger sample size may provide more reinforcing data and enhance the generalizability of the findings. Shear bond strength was only evaluated in the study after 5,000 thermocycles and a 24-hour water storage period. Shear bond strength was not evaluated for the long-term impacts of varying storage times, which may eventually affect durability. These conditions provide insight into initial shear bond strength; they do not serve as a definitive evaluation of bond longevity. Furthermore, elements like saliva and occlusal forces were not replicated, the shear bond strength testing method may not accurately reflect the clinical settings. Surface roughness may have an impact on adhesion results; therefore it should be studied in future studies. The study focused on only two types of bonding agents, but other bonding agents may yield different results, limiting the applicability of the findings to all available bonding systems.

## Conclusions

1. Ceramic brackets demonstrated superior shear bond strength compared to metal brackets, suggesting that material selection is crucial for optimizing orthodontic adhesion. Clinicians should consider using ceramic brackets when bonding to zirconia for improved long-term outcomes.
2. HEMA-containing adhesives resulted in higher shear bond strengths than HEMA-free alternatives, highlighting the importance of adhesive formulation in achieving reliable bonding outcomes. It is recommended that practitioners prioritize HEMA-containing adhesives for procedures involving zirconia to enhance bonding efficacy.
3. Storage settings were found to play a critical role in shear bond strength, yet they do not imply predictions of long-term performance based on the current study’s findings.

## Data Availability

During the current investigation, no datasets were created.
